# Effectiveness of intra-articular infiltration of platelet concentrates for the treatment of painful joint disorders in the temporomandibular joint: a systematic review

**DOI:** 10.4317/medoral.25658

**Published:** 2024-04-14

**Authors:** Daniela Lorena Quezada, Claudia Loreto López, Francisca Carolina Montini, Nicolás Patricio Skarmeta

**Affiliations:** 1 DDS. Orofacial Pain, Instituto de Cirugía y Especialidades Odontológicas, Las Condes, Chile; 2 DDS. Orofacial Pain, Bucall, Concepción Chile; 3 DDS, MSc. Universidad de los Andes, Chile; 4 DDS, MSc. Hospital del Salvador, SSMO, Providencia, Santiago, Chile; 5 DDS, MSc. OPH Clinic, Vitacura, Santiago de Chile

## Abstract

**Background:**

The role of Platelet-rich Plasma injections as a complementary therapy, together with other minimally invasive procedures, has been analyzed previously, however, there are no articles that evaluate the effects of intra-articular infiltration in the Temporomandibular Joint by itself. The aim of this article is to evaluate the effectiveness of intra-articular infiltration with Platelet-rich Plasma, as a single procedure, to both reduce pain and improve clinical parameters in painful joint disorders.

**Material and Methods:**

A systematic search was performed using the terms "Temporomandibular Joint Disorders" and "Platelet-rich plasma" in May 2021. Only the Clinical Trials found in the Pubmed/Medline, Embase, Cochrane Library/Cochrane CENTRAL, Google Scholar, and LILACS databases were selected.

**Results:**

Only four articles were selected for full-text review. Statistically significant differences were found in pain reduction Platelet-rich Plasma-based interventions with respect to preoperative measurements up to six months. Only two studies found significant intergroup differences favoring Platelet-rich Plasma over other interventions. In relation to maximum mouth opening, three studies reported an increase compared to the preoperative measurements.

**Conclusions:**

Platelet-rich Plasma might potentially be effective in reducing pain levels and improving clinical parameters such as interincisal distance. However, studies with better methodological quality, larger sample sizes, and lower risk of bias are required to assess the real value of this intervention in the management of painful joint disorders.

** Key words:**Platelet rich plasma (PRP), Painful Temporomandibular Joint Disorders, Minimally Invasive Procedures, Growth factors, Temporomandibular Joint Intra-articular injection.

## Introduction

Temporomandibular disorders (TMD) are a series of conditions that affect the temporomandibular joints, the masticatory muscles and/or the adjacent structures (1). Temporomandibular joint disorders (TMJD) are a subset of TMD characterized by producing pain in the preauricular region, limitation in mandibular movement and/or joint sounds (2). The etiology of TMJD is multifactorial and still uncertain since multiple factors may have an impact on altering the physiological balance of the system (3-6).

According to the First International Classification of Orofacial Pain (ICOP-1), painful joint disorders of the Temporomandibular Joint (TMJ) are categorized as primary when their etiology is unknown, or secondary when their origin is attribuTable to other conditions (e.g., alterations of the disc-condyle complex and/or osteoarthritis) (7).

The presence of inflammatory biomarkers has been associated with both joint degenerative processes and the report of joint pain. According to Ernberg (8), the presence of inflammatory mediators in synovial fluid shows high sensitivity in predicting the presence of joint pain, just as the absence of joint pain is highly specific in predicting lack of inflammatory activity. Additionally, recent studies have reported an increase in the expression of inflammatory cytokines and matrix metalloproteinases induced by these cytokines in patients with disc disorders and degenerative joint pathology (9-12).

In the management of painful TMJD, the use of various therapeutic strategies has been described, either by themselves or combined, including non-invasive therapies, pharmacological management, reversible occlusal therapies, intra-articular injections (e.g., hyaluronic acid (HA), corticosteroids (CS), autologous blood-derived products), arthrocentesis, and surgical procedures (2,13), among others.

Platelet concentrates have been proposed as a therapeutic option to manage joint inflammation and degenerative disorders, based on the high concentration of growth factors, such as: Platelet-derived Growth Factors (PDGFs), Transforming Growth Factor β1 and β2 (TGFβ1 and β2), Fibroblast Growth Factor (FGF), Vascular Endothelial Growth Factor (VEGF) and Insulin-Like Growth Factor (IGF). It has been hypothesized that these factors are involved in the stimulation of cell growth, modulation of the inflammatory response, and in the remodeling of the extracellular matrix and angiogenesis (14-16). Additionally, its potential effect on the restoration of intra-articular HA has been reported, enhancing its production by synoviocytes and increasing the synthesis of glycosaminoglycans by chondrocytes (17).

Although the effectiveness of intra-articular infiltration of platelet concentrates has been previously reviewed, interventions are commonly associated to other minimally invasive therapies or surgical procedures that include lysis and lavage of the joint, such as arthrocentesis or arthroscopic procedures (18-19).

According to the authors, there are no systematic reviews that evaluate the effects of PRP interventions by themselves. The objective of this systematic review is to assess whether intra-articular infiltrations of platelet concentrates in painful TMJ disorders are superior to placebo and other interventions to improve pain management and other clinical parameters.

## Material and Methods

This systematic review was carried out following the PRISMA statement reporting criteria. The research question was evaluated based on the PICOS criteria, where:

1. Population: Patients (humans) with painful joint disorders of the Temporomandibular Joint (i.e., arthralgia, arthritis, osteoarthritis, or joint diseases according to DC/TMD criteria, equivalent diagnoses, or diagnosis confirmed by images).

2. Intervention: Intra-articular injections of platelet concentrates in single-administration or in a series of administrations into the TMJ (PRP or Plasma Rich in Growth Factors PRGF) without any additional interventional procedure (e.g. TMJ arthrocentesis or arthroscopy)

3. Comparison: arthrocentesis by itself, arthroscopy by itself, intra-articular infiltration therapies such as normal saline, lactated Ringer's serum, HA (e.g. low-molecular-weight, moderate-molecular-wight, or high-molecular-weight) and/or CS (e.g. betamethasone, methylprednisolone, triamcinolone hexacetonide, etc.) injections in single or multiple administrations.

4. Outcome: Primary: the decrease in baseline pain values using the Visual Analogue Scale (VAS) or the decrease of in baseline pain values using other scales; Secondary: gain in maximum mouth opening (MMO) or interincisal distance, other measures of ranges of motion, masticatory efficiency.

5. Setting: private clinical centers, public or private clinical hospitals, university clinics.

- Inclusion and exclusion criteria

The search criteria were limited to randomized clinical trials or prospective controlled clinical trials in humans, using clinical diagnostic criteria with validated clinical criteria such as DC/TMD or non-validated diagnoses corroborated using magnetic resonance imaging (MRI) or cone beam computerized imaging tomography (CBCT). Intra-articular infiltration protocols that were applied in mono or multiple administration were included. All those studies that did not meet the inclusion criteria or used additional interventions combined with the application of platelet concentrates (e.g., arthroscopy + PRP or arthrocentesis + PRP) were excluded.

- Search criteria

The search protocol was carried out following the guidelines of the Cochrane Handbook for Systematic Reviews (version 6.2) and using the RevMan software (version 5.4). In October 2020, a pilot search was carried out by the authors DLQ and CLL, without doing data extraction, with the aim of identifying potentially relevant studies. The pilot search was carried out using the MeSH terms: “Temporomandibular Joint Disorders”; “AND; "Platelet-Rich Plasma" where 137 studies were identified in various databases.

In early May 2021, a new search was conducted in English and Spanish with no specific date of inclusion (authors DLQ and CLL). The English language search was performed using the MeSH terms “Temporomandibular Joint Disorders”, “AND”, and “Platelet-rich plasma” in the Pubmed/Medline, Embase and Cochrane Library/Cochrane CENTRAL databases. For the rest of the process, filters such as: "human" and "trials" were applied, with the aim of increasing the precision of the search. For the search in Spanish, the LILACS database was used, using the DeCS terms “Temporomandibular Joint Disorders” and “Platelet Rich Plasma”. At the end of May 2021, an extension of the search was carried out with the aim of identifying gray literature in Google Scholar, as well as the reference review of the studies that were selected.

- Data Selection and Analysis

The study selection was carried out by the authors DLQ and CLL based on the inclusion and exclusion criteria and guided by the author FCM. The studies were selected in the following order: Title, Abstract and Full Text. Discrepancies regarding selection were settled by the author FCM. The study selection was initially carried out in Google Forms, to later be entered in RevMan 5.4. The data extraction was performed by DLQ and CLL, extracting Authors, Year, Design and Study Type, Study Population (patient number, age, gender), and Measurements (descriptive measurements and quantitative data, *p value*, means, among others). The Tables, as well as the bias assessment of the studies, were carried out by the authors NPS and FCM using RevMan 5.4 based on the Cochrane Handbook for Systematic Reviews.

The authors (NPS and FCM) assessed the quality of the included studies and identified potential risks of bias using Cochrane's risk-of-bias tool for randomized trials (RoB2). Critical domains were evaluated, including random sequence generation, allocation concealment, blinding of participants and outcome assessors, incomplete outcome reporting, and selective reporting of outcomes. This comprehensive assessment enabled categorization of each domain into low risk of bias, some concerns, or high risk of bias, thus determining the overall quality and potential limitations of the studies.

- Protocol register

This systematic review was registered in the International Prospective Register of Systematic Reviews in Health and Social Care of the UK National Institute for Health Research; “PROSPERO”: CRD42021267718.

## Results

- Study selection

A total of 88 potential articles were identified using search criteria. Of these, 30 were found through database searches such as Pubmed, Cochrane Library, Embase, and Lilacs, while 58 were identified through gray literature searches, Google Scholar, and reference checks. After removing duplicate articles, the titles and abstracts of 74 remaining articles were reviewed for potential inclusion.

**Figure 1:**
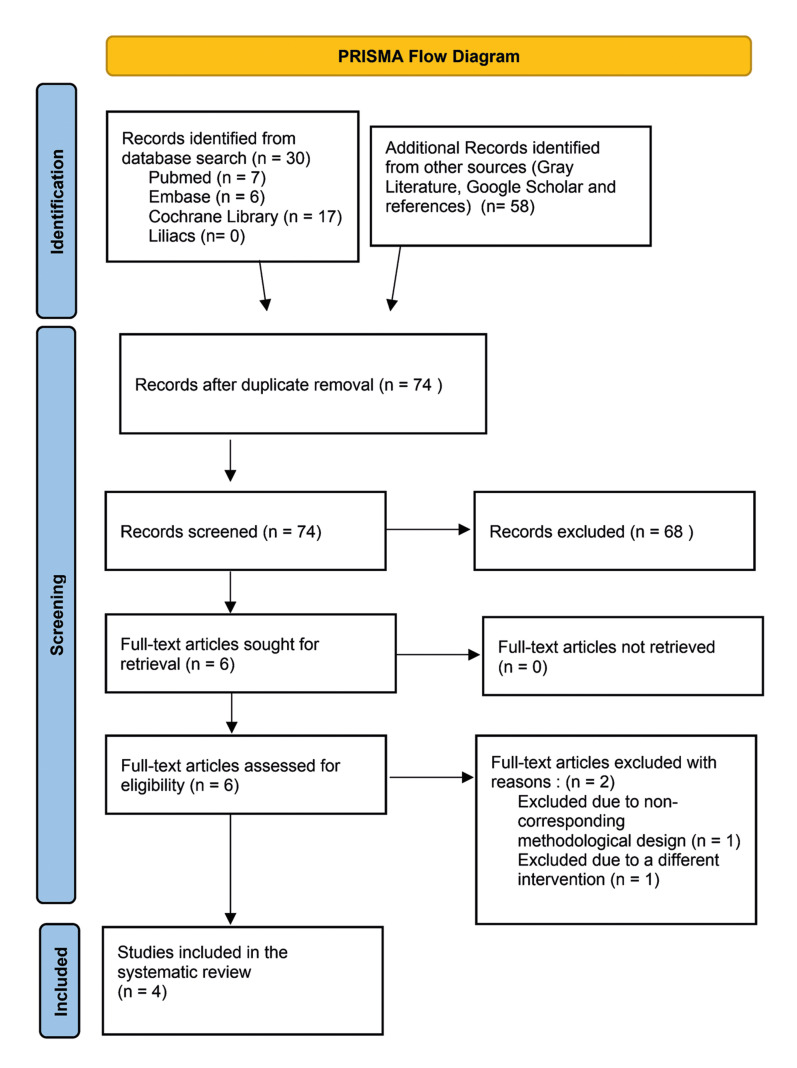
Flowchart diagram for selection eligible studies.

During the review process, several exclusions were made for various reasons. Eight studies in the development phase were excluded, either due to non-conforming interventions or incomplete information. Eighteen studies were excluded for having inappropriate methodological designs, such as observational studies, case series, reports, editorials, narrative reviews, and systematic reviews. Sixteen articles were excluded for not studying the target population, while 23 studies were excluded for combining PRP with other interventions. Six more studies were excluded because they were not written in English or Spanish. See the flow chart (Fig. 1).

**Figure 2:**
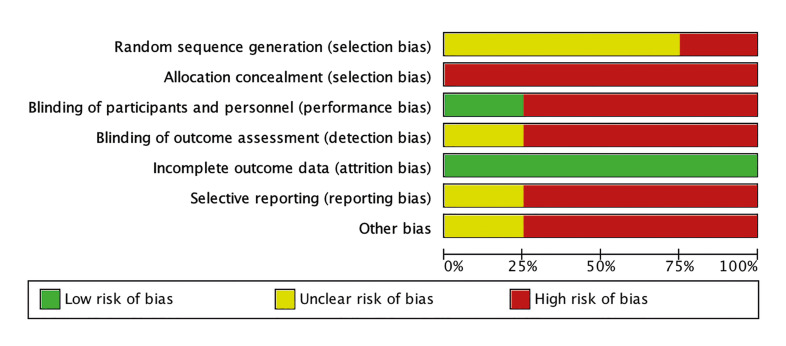
Graphic representation of risk analysis.

**Figure 3:**
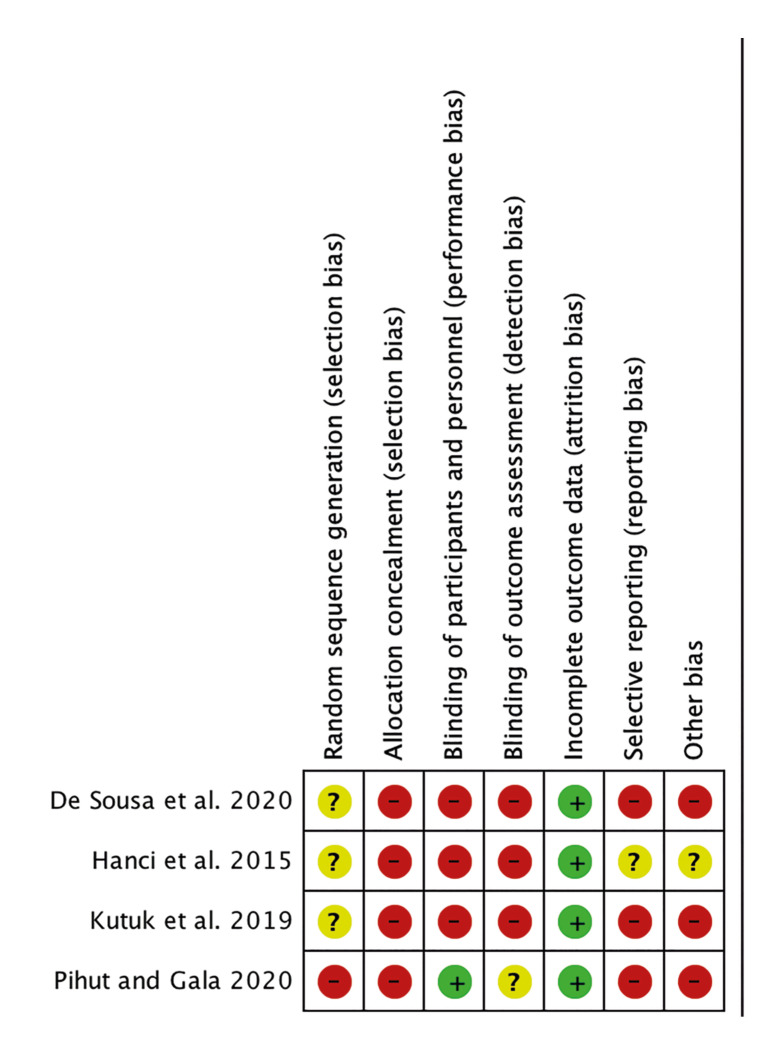
Summary of risk of bias.

Ultimately, six studies were chosen for full-text analysis (20-25). Among them, two were excluded: one for not presenting an appropriate methodological design (case series) and the other for including an intervention that combined arthrocentesis with PRP. The remaining four studies (20,23-25) were included for data extraction, qualitative analysis (Fig. 2), and bias analysis (Fig. 3). Table 1 and Table 2 provide detailed information about these included studies.

- Study characteristics

In the included studies, 260 patients participated, with 105 receiving PRP infiltrations and 155 receiving control procedures or alternative intra-articular treatments. The patients had a mean age ranging from 27.2 to 43.1 years, with a higher representation of females than males, as shown in Table 1 and Table 2.

All the studies reported a statistically significant reduction in pain levels compared to preoperative baseline VAS values after PRP-based intra-articular treatments (20,23-25). Hanci *et al*. (24) observed a substantial reduction in post-operative VAS values over the long term after a single PRP administration.

**Table 1:**
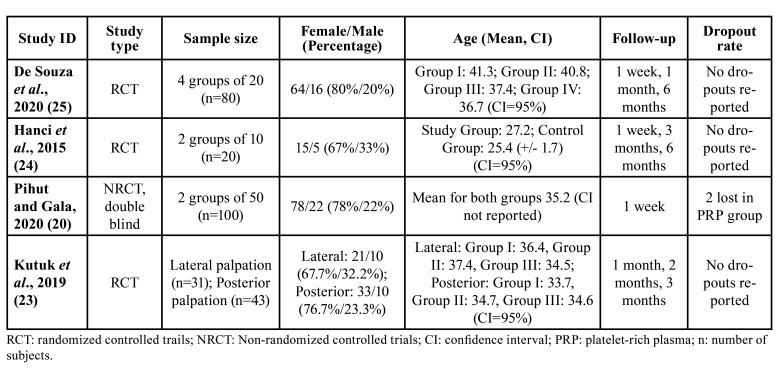
Demographic and clinical characteristics of the included studies.

**Table 2:**
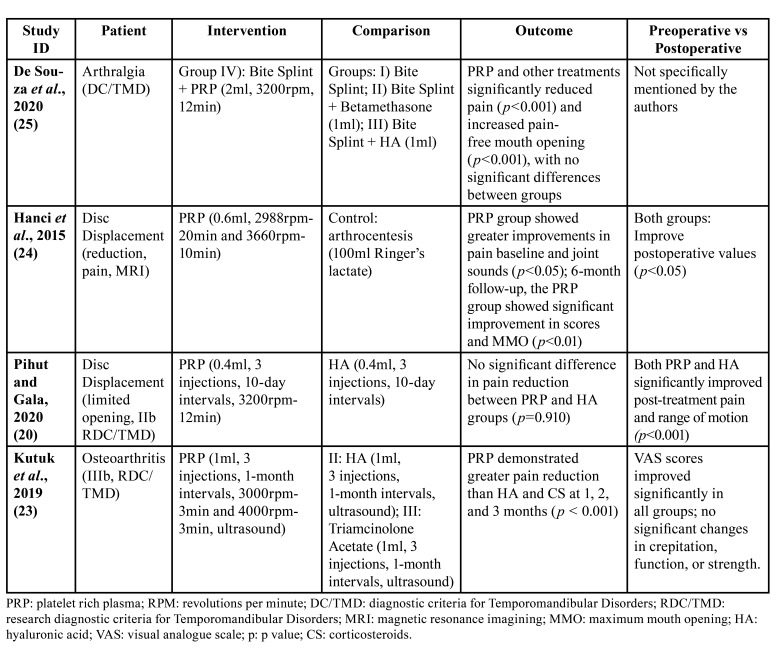
Included studies according to PICOS criteria.

The most significant decrease was noted between pre-injection (6.69 ± 2.21) and 6 months post-injection (0.07 ± 0.27; *p* = 0.01). Similarly, Kutuk *et al*. (23) found significant pain alleviation after a series of PRP injections relative to injections with HA or CS, with noTable improvements at 1-month (*p* < 0.001), 2 months (*p* < 0.001), and 3 months (*p* < 0.001) follow-ups. De Souza *et al*, found that there was a significant reduction in pain (*p*< 0.001) from the baseline to the 6-month follow-up in the group treated with PRP and bite splint (BS). Lastly, Pihut and Gala documented a pronounced decrease in pain in the PRP group, finding a statistically significant improvement in post-treatment VAS scores (*p* < 0.001).

Secondary outcomes showed improvements in range of motion (MMO) and joint functionality. De Souza *et al*. observed increased pain-free mouth opening (25); Hanci *et al*. reported MMO improvement at 6 months in the PRP group (*p*< 0.01) and improved joint sounds (24); Pihut and Gala found improvements in both PRP and HA groups' range of motion (*p* < 0.001 within each group) (20). Kutuk *et al*. (23), observed improvements in crepitation, loss of function, and loss of strength in the PRP group, but these were not statistically significant in comparison to the HA and CS groups.

A meta-analysis was not performed due to the significant heterogeneity among studies, stemming from variations in study designs, interventions, comparison groups, and outcome measures, as well as the high risk of bias.

Upon conducting a bias analysis, all the studies exhibited a high risk of bias due to unclear randomization methods and inadequate allocation concealment. (Fig. 2, Fig. 3) In these studies, it was unclear if the participants were randomly assigned to the treatment groups, leading to a potential selection bias. Additionally, the lack of blinding in most of the studies could have influenced the results, as participants and outcome assessors may have had prior knowledge of the treatment received.

## Discussion

In this systematic review, all included studies reported that PRP infiltrations led to a statistically significant reduction in pain compared to preoperative baseline measurements (20,23-25), affirming PRP's overall effectiveness. However, only two studies (23,24) identified a statistically significant difference in pain reduction favoring PRP over other interventions. This indicates that while PRP is consistently effective compared to baseline, its superiority over other treatments requires further validation.

The studies of De Souza *et al*. (25) and Hanci *et al*. (24) compared two different PRP single-administration protocols against various interventions and controls. De Souza *et al*. reported that all treatment groups effectively reduced pain compared to the control (*p* < 0.001). However, they found no significant difference in the reduction of pain intensity among the treatment groups (*p* = 0.077). Conversely, Hanci *et al*. (24) reported that patients receiving PRP intervention experienced a more substantial reduction in pain compared to those undergoing conventional arthrocentesis, with the differences being statistically significant (*p* < 0.05).

Pihut and Gala(20) and Kutuk *et al*.(23) compared a series of three consecutive PRP infiltrations. The first authors compared consecutive TMJ infiltrations of PRP and HA every ten days, not finding a statistically significant decrease in the VAS between both groups (*p* =0.910).

Kutuk *et al*. (23) reported that a monthly series of three PRP infiltrations showed superior results in reducing pain during lateral palpation for patients with arthralgia compared to HA and CS infiltrations. Notably, at the first month, the PRP group experienced a greater decrease in VAS scores than the HA group (*p* < 0.001). This trend continued at the second and third month follow-ups, with the PRP group showing more substantial reductions in VAS levels relative to both the HA and CS groups, affirming the sustained efficacy of PRP over time (*p* < 0.001).

Regarding secondary outcomes, only three studies measured the interincisal distance (20,24,25); one measured sound in decibels (24); and one measured crepitation, loss of strength, and loss of function without detailing how the measurements were made. (23)

The MMO increased in all three studies that performed this measurement. In addition, Hanci *et al*. reported a statistically significant decrease in noise in decibels in conjunction with a significant improvement in MMO.(24)

To the authors’ knowledge, there are no published reviews evaluating the role of PRP as a single intervention. However, several publications exist evaluating the role of PRP combined with other interventional procedures such as arthrocentesis or arthroscopy. (19,26-28) Similarly to our results, one systematic review and two meta-analyses showed favorable results towards using PRP protocols in combination with arthrocentesis or arthroscopy. (19,26,27) Nonetheless, as suggested by Haigler *et al*. (19), it is plausible that many of the studies included in these systematic reviews presented performance and detection biases, affecting the validity of the conclusions. Another systematic review and meta-analysis by Al-Hamed *et al*. (28) that evaluated the effectiveness of PRP and PRGF co-administrated with arthroscopy or arthrocentesis reported that both the study and control groups presented a significant improvement in the VAS baselines. Also, four out of five studies showed greater effectiveness of platelet concentrates over HA; three studies showed statistically significant differences favoring the effect of platelet concentrates over saline infiltrates, arthrocentesis, or arthroscopy on their own.

The four articles included in this systematic review effectively reduced pain levels. However, since the included studies were highly heterogeneous in their administration protocols, how the platelet concentrates were prepared, and which painful TMJD were evaluated, these results must be analyzed with caution. Moreover, the main limitation of this review is that only a few studies met the inclusion criteria, all of them having relatively small sample sizes and a high risk of bias.

## Conclusions

The analyzed studies in this systematic review, based solely on intra-articular infiltrations with PRP, showed effectiveness in reducing pain and increasing interincisal distance up to six months after their administration. However, these findings must be analyzed with caution since the included studies were highly heterogeneous, had small sample sizes, presented a high risk of bias, preparation protocols presented variations and PRP administration and the diagnoses varied greatly among the included studies. The authors’ conclusion is that there is not enough evidence to support the effectiveness of PRP as a sole intervention. Better quality RCTs, with larger numbers of participants, are needed to assess the real value of this intervention in the management of painful TMJD, designed specifically with the aim of reducing the risk of bias.
